# *Sneathia amnii* bacteraemia and chorioamnionitis leading to second trimester abortion: a case report

**DOI:** 10.1099/acmi.0.000290

**Published:** 2021-12-09

**Authors:** Lien Gruwier, Aaron Sprenkels, Sofie Hulsbosch, Anne Vankeerberghen, Reinoud Cartuyvels

**Affiliations:** ^1^​ Jessa Hospital, Laboratory of Clinical Microbiology, Hasselt, Belgium; ^2^​ Jessa Hospital, Department of Gynaecology, Hasselt, Belgium; ^3^​ OLV hospital, Laboratory of Clinical Microbiology, Aalst, Belgium

**Keywords:** 16S rRNA gene sequencing, blood culture, septic abortion, *Sneathia amnii*

## Abstract

**Background:**

*
Sneathia amnii* (formerly designated as *Leptotrichia amnionii *) was first described in 2002 in the USA. Members of the genus *Sneathia* can be part of the normal flora of the genitourinary tract, but have been implicated in invasive (mostly gynaecological) infections.

**Case presentation:**

To the best of our knowledge, here we present the first case of *S. amnii* infection in Belgium, in a young woman presenting with fever leading to second trimester septic abortion.

**Conclusion:**

Despite its pathogenicity, *S. amnii* remains an underrated cause of infections due to inherent difficulties with conventional laboratory methods. By extracting the bacterial DNA directly from the blood culture broth and performing a 16S ribosomal RNA gene sequence analysis we succeeded in identifying *S. amnii* as the most probable cause of the septic abortion in our patient.

## Background

*Sneathia amnii* is a fastidious, non-motile, pleomorphic Gram-negative (cocco)bacillus that grows anaerobically on blood agar after approximately 3 days of incubation [1]. The bacterium was first isolated in 2002 from the amniotic fluid of a woman after intrauterine foetal demise [2]. Species of *Sneathia* are a part of the normal microbiota of the genitourinary tracts of men and women, but they are also associated with a variety of clinical conditions, including bacterial vaginosis, pre-eclampsia, preterm labour, spontaneous abortion, peripartal bacteraemia and other invasive infections. Some authors suggest a role of *Sneathia* species in the pathogenesis of sexually transmitted diseases and cervical cancer [3, 4].

## Case presentation

### Clinical approach

A young woman, gravida 2 para 1, presented to the emergency department at 13 weeks and 5 days of gestation with complaints of high fever and left flank pain. Her current pregnancy was uneventful except for postcoital bleeding 1 day before presentation. The patient was an active smoker. She did not take any medication except pregnancy-related supplements. Her medical history consisted of gastric bypass surgery 7 years earlier.

On admission, the patient’s blood pressure was 116/85 mmHg, her heart rate was 89 beats min^−1^ and her temperature was 39.0 °C. The patient appeared unwell, but was alert and fully aware. Her abdomen was soft on palpation. A speculum examination showed neither significant bleeding nor purulent discharge from the cervical os. Laboratory tests showed a normal leucocyte count of 7.63×10^9^ l^−1^, haemoglobin of 11.5 g dl^−1^, a platelet count of 192×10^9^ l^−1^, a moderately elevated CRP (48 mg l^−1^) and normal renal and liver function tests. Two sets of blood cultures were drawn and a midstream urine sample was sent to the laboratory. Abdominal ultrasonography was negative for intra-abdominal pathology and revealed an intrauterine pregnancy with heart movement. The working diagnosis was acute pyelonephritis for which empirical IV amoxicillin/clavulanic acid (4 g per day) was administered.

On the second day of hospitalization, abdominal ultrasonography revealed intrauterine foetal death. Vaginal misoprostol was administered for the induction of labour. After 2 days and the insertion of a transcervical balloon catheter expulsion of the foetus followed.

One of the four blood culture bottles taken at admission became positive at day two for Gram-negative bacilli, though without a reliable identification of the causative species. Amoxicillin/clavulanic acid was discontinued and treatment with IV amoxicillin (8 g per day) in combination with IV gentamycin (0.24 g per day) was initiated by the treating gynaecologist because of suspected pelvic inflammatory disease. The patient’s condition gradually improved and she was discharged without sequelae or complaints 5 days after admission. Pathological analysis of the placenta revealed severe chorioamnionitis and villitis. On a follow-up visit 5 weeks after discharge she was clinically well.

### Microbiological investigations

Automated microscopic analysis of the urine sample by the sediMAX conTRUST (A. Menarini Diagnostics) revealed a slightly elevated white blood cell count (43 WBC µl^−1^). The urine specimen was inoculated onto a MacConkey agar and a Columbia agar with 5 % sheep blood (BD)*,* but only normal urogenital flora was grown after 24 h of incubation.

The two sets of blood cultures that were drawn at admission were incubated in the BacTec device (BD). Three out of four blood culture bottles remained sterile after 7 days of incubation. However, in one (aerobic) bottle BacTec alerted growth after 29 h of incubation. Gram staining of the positive bottle revealed long Gram-negative rods. A specimen from the positive blood culture bottle was inoculated onto MacConkey agar, Columbia agar with 5 % sheep blood, chocolate agar, Schaedler agar and a thioglycollate broth, and incubated at 35 °C under 5 % CO^2^ and anaerobic atmosphere. A culture from the sample remained sterile after 20 days of incubation.

A molecular approach was undertaken to allow identification of the causative micro-organism. Bacterial DNA was extracted from 1.5 ml of blood culture broth from the positive bottle. First, the broth was carefully mixed and centrifuged at 6400 ***g*** for 10 s. The supernatant was transferred to another tube and centrifuged at 20 800 ***g*** for 1 min. The pellet was resuspended in 1 ml physiological water. After another centrifugation step, the pellet was resuspended in 500 µl TE buffer and heated at 95 °C during 15 min. The bacterial 16S rRNA gene was amplified using a forward primer (5′-AGT TTG ATC CTG GCT CAG-3′) and a reverse primer (5′-GTA TTA CCG CGG CTG CTG-3′). PCR mixtures (90 µl) contained 10 µl PCR buffer, 2 µl 10 µM of each primer, 3 µl 50 mM MgCl_2_, 10 µl 2 mM dXTPs, 1 µl TaqDNA polymerase (5 U µl^−1^) (Life Technologies) and 62 µl PCR-grade water. Then 90 µl of the PCR mix, together with 10 µl of extracted DNA, was amplified with the Gene Amp PCR system 2400 (Applied Biosystems). PCR conditions were: 95 °C for 5 min; 3 cycles of 95 °C for 45 min, 50 °C for 2 min and 72 °C for 1 min; 30 cycles of 95 °C for 20 min, 50 °C for 1 min and 72 °C for 1 min; with a final elongation step of 72 °C for 7 min. Next, a semi-nested PCR was executed following the same protocol as described above, except for the use of a second reverse primer (5′-ACTGCTGCCTCCCGTAGGAG-3′). Afterwards, the amplicon was purified using the Qiaquick PCR Purification kit (Qiagen). PCR products were sequenced by capillary electrophoresis (GenomeLab GeXP Genetic Analysis System, Beckman Coulter) and species identification was based on identity with 16S rDNA sequences in the National Center for Biotechnology (NCBI) database. blast analysis identified the isolate as ‘* Sneathia amnii *’ with a maximal identity of 100 % *for ‘S. amnii*’ (multiple strains).

## Full-Text

## Discussion

Various reports have demonstrated that *Sneathia* species can be a component of the normal urogenital microbiome, but are also associated with sexually transmitted infections (STIs), bacterial vaginosis (BV) and cervical cancer. As part of the Vaginal Human Microbiome Project, Harwich *et al*. detected *Sneathia* species in 43.3 % of mid-vaginal samples taken from women in outpatient clinics in the USA. Both *Sneathia sanguinegens* and *S. amnii* were detected, but the majority of *Sneathia* reads (76.3 %) classified to *S. amnii* [4]. In 2010, Nelson *et al*. investigated the microbiome of the male urogenital tract and found that men can also be colonized with *Sneathia* species. Using 16S rRNA gene sequencing, they discovered that the urine microbiomes from men with STIs were dominated by fastidious, anaerobic bacteria (including *Sneathia* species), while the same taxa were rare in STI-negative individuals. However, the study could not distinguish whether the STI-associated communities precede, are co-transmitted with, or are established subsequent to the STI [5]. Also in 2010, Nawrot *et al*. studied the rate of *Sneathia* colonization in female subjects with human papillomavirus (HPV). Although there was no positive correlation between HPV and *Sneathia *, there was a significant correlation between colonization with *S. amnii* and cervical cancer in HPV-positive subjects [6]. Other studies investigating the bacterial taxa associated with BV have revealed an association between BV and the presence of *Sneathia* species [7–9]. More research is still needed to clearly define the role of these bacteria in the aetiology and pathology of BV.

Several case reports (including ours) have proven the pathogenicity of *S. amnii* in invasive (mostly gynaecological) infections. *S. amnii* has been associated with serious obstetric complications, including peripartal bacteraemia [10] and chorioamnionitis, leading to spontaneous abortions and preterm labour [1–3, 10–16]. *S. amnii* has been described as the causative agent of endometritis, salpingitis, tubo-ovarian abscess and postpartum renal abscess [8, 12, 17, 18]. Although the virulence factors that promote uterine and intra-amniotic invasion remain largely unknown, it has been suggested that enzymes (including sialidase) could play a role in hydrolysis of the cervical mucus [4]. Recently, a cytotoxic exotoxin produced by *S. amnii* was identified and isolated. This cytotoxin lyses human red blood cells and is capable of permeabilizing chorionic trophoblasts and thus may play an important role in its virulence [19].

One study among renal transplant recipients describes the identification of *S. amnii* in a urinary tract specimen of a female non-pregnant patient with leukocyturia, raising the suspicion of *S. amnii* as aetiological agent of urinary tract infections [20]. Reported cases of early-onset neonatal meningitis and epidural abscess (1 week following hysteroscopy) demonstrate that *S. amnii* has the potential to cause infections outside of the reproductive tract [21, 22]. A case of septic arthritis in an immunocompromised man revealed that the organism is not restricted to the female genital tract [23].

Due to its complex growth requirements, *S. amnii* is an underappreciated cause of infections. This fastidious species grows preferentially under anaerobic atmospheric conditions on chocolate agar plates after several days of incubation [2]. Its growth rate can be enhanced by the addition of human serum [4]. *S. amnii* is poorly characterized using routine phenotypic laboratory tests due to its poor chemical reactivity. At present no conclusive identification of the strain has been be achieved with matrix-assisted laser desorption/ionization-time of flight mass spectrometry (MALDI-TOF MS), resulting in the use of molecular techniques that are more time-consuming, expensive and technically demanding [21].

Harwich *et al*. performed sensitivity assays for a number of antibiotics, using a broth dilution method: *S. amnii* exhibited relatively high levels of resistance to tetracycline (>50 µg ml^−1^) and ciprofloxacin (>25 µg ml^−1^) and a very low level of resistance to metronidazole (0.5 µg ml^−1^) [4]. Decroix *et al*. determined sensitivity using agar diffusion (Etest, AB Biodisk) on chocolate polyvitex agar. As the interpretive breakpoints for *Sneathia* have not yet been established, they made use of the Clinical and Laboratory Standards Institute (CLSI) breakpoints for anaerobic organisms other than the *Bacteroides fragilis* group. It revealed that their *S. amnii* isolate was highly susceptible to penicillin G, amoxicillin, cefotaxime, ceftriaxone and metronidazole [20]. Because the numbers of reported cases are limited, the preferred antibiotic for the treatment of S. *amnii* infection remains questionable. So far, most infections caused by *S. amnii* have been treated successfully with amoxicillin/clavulanate or metronidazole [12, 23].

Our case report has some limitations. Even after 20 days of anaerobic (and aerobic) incubation, there was no growth of *S. amnii* on traditional agar plates. The fastidious nature of *S. amnii* has been documented extensively and only just over half of the previous case reports describe growth after prolonged incubation under anaerobic atmospheric conditions. Other than the complex growth requirements of *S. amnii *, we do not have a clear explanation for the lack of growth on our commercial agar plates. Since the clinical picture of the patient, the pathological analysis of the placenta and the Gram stain of the blood culture were consistent with the reported pathogenicity and microscopic appearance of *S. amnii *, we concluded that there was enough evidence to consider *S. amnii* as the most probable cause of the septic abortion [4]. However, since there was no microbiological or molecular analysis of (placental) tissues we lack solid evidence that links the presence of *S. amnii* in the bloodstream to the clinical outcome of the patient.

## Conclusions

*Sneathia amnii* is an emerging opportunistic pathogen that can be part of the normal vaginal flora and that is mainly associated with infections of the genital tract. However, cases of neonatal meningitis, epidural abscess and septic arthritis demonstrate that *S. amnii* has the potential to cause infections outside of the reproductive tract as well.

Despite its pathogenicity, *S. amnii* remains an underrated cause of infections due to inherent difficulties with conventional laboratory methods. In most cases, culture demonstrates anaerobic growth of pleomorphic Gram-negative (cocco)bacilli, after which 16S ribosomal RNA sequencing is used to determine the species identification. Our case was exceptional, since there was no growth on traditional agar plates after 20 days of anaerobic incubation. By extracting the bacterial DNA directly from the blood culture broth and performing a 16S ribosomal RNA sequence analysis we succeeded in identifying *S. amnii* as the most probable cause of the second trimester septic abortion in our patient.

### Availability of data and materials

The datasets used and/or analysed during the current study are available from the corresponding author on reasonable request.
